# Neglected Avian Blood Parasites (Onchocercidae and Trypanosomatidae) in Migratory Passerines of the Temperate Zone, Eastern Baltic Region

**DOI:** 10.3390/pathogens14050452

**Published:** 2025-05-05

**Authors:** Rasa Bernotienė, Tatjana Iezhova, Vytautas Eigirdas, Vytautas Jusys, Margarita Kazak, Rasa Binkienė

**Affiliations:** 1 State Scientific Research Institute Nature Research Centre, Akademijos 2, 08412 Vilnius, Lithuania; tatjana.jezova@gamtc.lt (T.I.); margarita.kazak@gamtc.lt (M.K.); 2 Ventės Ragas Ornithological Station, Marių 24, 99361 Ventė, Lithuania; v.eigirdas@gmail.com (V.E.); vrventragis@gmail.com (V.J.)

**Keywords:** diet, filaria, juvenile, nematodes, nesting, trypanosomes

## Abstract

Passerine birds (n = 3335) of 19 species were caught and investigated for the presence of Trypanosomatidae and Onchocercidae parasites using the buffy coat method, microscopy and PCR in Ventės Ragas, Lithuania. Data on the spread patterns of these parasites are still lacking. The prevalences of *Trypanosoma* parasites in birds of different species varied from 2.2% to 36.1%, while the prevalences of Onchocercidae parasites varied from 0% to 17.3%. Statistically significant differences between spring and autumn in the prevalences of Trypanosomatidae were determined for *Acrocephalus schoenobaenus, Hirundo rustica* and *Turdus philomelos.* No significant differences between the prevalences of Onchocercidae in spring and autumn were determined. The prevalence of *Trypanosoma* was significantly higher for long-distance migrant birds compared with short-distance migrants, for omnivorous birds compared with insectivorous birds, and for open-nesting birds compared with birds nesting in nest boxes. The prevalences of Onchocercidae parasites did not differ for the same bird groups except for the prevalence in omnivorous birds, which was higher compared with insectivorous birds. Both groups of parasites were detected in juveniles, showing the presence of transmission in the study area. The diet, breeding behaviour and migration features of avian hosts can influence the prevalence of avian blood parasites.

## 1. Introduction

Birds are distributed globally and are suitable hosts for all groups of parasites [1]. Avian blood parasites are detected worldwide except for in Antarctica [2,3,4,5]. They have to rely on certain insect vectors to carry them from bird to a bird and, in this way, have a complex life cycle with at least two different hosts [6]. Parasites influence species’ coexistence and extirpation by altering competition, predation and herbivory, and these effects can influence ecosystem properties [1]. Some avian blood parasites, such as haemosporidians, are well studied because they are known to cause mortality and morbidity to their hosts especially in naïve bird populations [7,8,9]. Some avian blood parasites such as Onchocercidae (Nematoda) and Trypanosomatidae (Euglenozoa) are neglected due to the low economic importance of their host species [10] or the lack of knowledge about their effects on host health. Data are particularly lacking on wild bird populations, whose pathologies are often overlooked because parasite-affected birds can be less active and therefore do not fall into bird-catching nets or are easily caught by predators [11].

Filarioid Onchocercidae nematodes are found in the tissues and tissue cavities of many vertebrate groups and their microfilariae are present in the blood stream or skin [4,6]. Sixteen genera of filariae are parasitising in birds. The filarial species of *Aproctella* Cram, 1931, *Andersofilaria* Bartlett and Bain, 1987, *Cardiofilaria* Strom, 1937, *Chandlerella* Yorke and Maplestone, 1936, *Eufilaria* Seurat, 1921, *Paronchocerca* Peters, 1936, *Pelecitus* Railliet and Henry1910, *Pseudlemdana* Sonin and Shumilo, 1964 and *Splendidofilaria* Skrjabin, 1923 are found in passerines [12,13,14,15,16,17,18,19,20,21,22]. Recent studies of onchocercidian parasites revealed six species of Passeriformes birds to be infected with filarioid nematodes in the eastern Baltic region: *Acrocephalus scirpaceus* (Hermann, 1804) was infected with *Eufilaria acrocephalusi* Binkienė, 2021; *Sylvia borin* (Boddaert, 1783) with *Eufilaria sylviae* Binkienė, 2021; *Sylvia atricapilla* (L.) with *Splendidofilaria bartletti* Binkienė, 202; *Linaria cannabina* (L.) with *Chandlerella sinensis* Li, 1933; and *Turdus merula* L. and *Turdus philomelos* Brehm, 1831 with *Splendidofilaria mavis* (Leiper, 1909) [14,18,23]. The vectors of these parasites are not well known but the majority of these parasites are thought to be transmitted by the bite of bloodsucking insects [6].

Despite the fact that it is hard to find a bird population not infected with *Trypanosoma* Gruby, 1843 parasites [24], avian trypanosomes are still being neglected—although some data are present about the mortality of birds caused by these parasites [25]. Three paraphyletic groups named after principal species of *Trypanosoma* are found in birds in temperate zones: the *T. bennetti*-*everetti* group (hereafter *T. bennetti* group) of so-called small-size (the mean length without free flagellum in the blood stream is 17.4 µm) trypanosomes [26] which are found in passerine and raptor birds and are transmitted by *Culicoides* vectors [27,28]; the *T. culicavium-corvi* group of large-size trypanosomes (hereafter *T. culicavium* group) obtained from passerine birds and dipteran vectors; and the *T. avium-thomasbancrofti* group (hereafter *T. avium* group) of large-size trypanosomes (mean length 39.8–52.6 µm) isolated from passerines [29], raptor birds and several dipteran vectors [10,27]. Although trypanosomes are transmitted by bloodsucking insects, the mode of transmission is different from that of filarial infection. Avian trypanosomes are transmitted not by insect bite, but by eating an infected insect or via conjunctiva [30]; therefore, the patterns of spread of these two parasite groups may differ.

Conditions that may be significant for the spread of parasites and their prevalence in vertebrate hosts need to be evaluated; this would help to predict the spread of new pathogens and the diseases they cause. One of the main factors affecting parasite transmission is the frequency of contact between hosts and vectors [31]. Bird migration increases the contact opportunities with new vectors and new parasites and the chances of getting diseases not only at breeding grounds but also at wintering sites [32]. The spreading of avian parasites with migratory birds can have an influence on the functioning of the entire ecosystem, as parasites carried to other regions can affect local and naïve host populations [7,8]. The introduction of new parasites into the ecosystem can also affect vector populations, as some parasites have been shown to cause vector mortality [23,33,34]. It is important to emphasise that the characteristics of migration of different bird species differ, as long-distance migrants (those that migrate south of the Mediterranean Sea) and short-distance migrants (those that do not migrate south of the Mediterranean Sea) can be distinguished [35,36]. The infection rates can be influenced by the bird diet as in the case of *Trypanosoma* parasites transmission can take place via ingestion of the infected insect. Nesting in open nests makes birds more accessible to vectors compared with nesting in nest boxes or tree hollows, as some insects avoid biting inside or in the dark [37].

The aim of this study was to evaluate infection with Onchocercidae and *Trypanosoma* parasites in passerine birds in spring and in autumn and to analyse the trends of infection with the migration characteristics, diet and nesting habitats of birds. Based on infection rates in juveniles, we aimed to assess the possibility of parasite transmission in the study area.

## 2. Materials and Methods

### 2.1. Bird Collection, Blood Fixation and Staining

In total, 3335 Passeriformes birds belonging to 19 species (Table 1) were caught at Ventės Ragas Ornithological Station, Lithuania (55°20′28.1″ N 21°11′25.3″ E), during spring (April–May) and autumn (September–October) in 2018–2024 (Supplementary Table S1). Birds were captured with mist nets, zig-zag traps and large funnel-type traps. They were ringed, identified and examined at the study site.

Blood (about 20–50 µL, depending on the size of the bird) was taken from the *vena cutanea ulnaris*. The first few drops of fresh blood were used to prepare two thin blood films per specimen, which were used for morphological examination, if necessary. Second, approximately 10 μL of blood was fixed in SET buffer (0.05 M tris, 0.15 M NaCl, 0.5 M EDTA, pH 8.0) for PCR-based analysis of trypanosomes. For the detection of *Trypanosoma-* and microfilariae-infected individuals, the remaining blood was used by the buffy coat method [38]. The method is based on the centrifugation of blood in the capillary and the resulting separation of blood parasites in the layer between blood cells and plasma. This method allows us to identify *Trypanosoma* parasites to the *Trypanosoma* group level (small or large trypanosomes or their mixed infection). From microfilariae-positive birds, additionally, approximately 20–30 μL of blood was taken for the preparation of several additional blood films (for collection purposes) and the remaining blood (approximately 10-20 µL) was fixed in 95% ethanol for the molecular diagnosis of microfilaria. All blood films were rapidly air-dried using a battery-powered fan, fixed in absolute methanol. Whole-blood films were examined at low magnification (×200); if microfilariae were present, the parasites were studied at medium (×500) and high (×1000) magnifications, and their images were prepared. Measurements and photomicrographs of live and stained with Giemsa nematode larvae were carried out using a digital image analysis system (DeltaOptical DLTCam Viewer 3.7.8301).

Microfilariae were first identified morphologically based on length, width, shape of tale and distances from the anterior part to the nerve-ring, excretory pore, inner body and anal pore [4,14,20,21]. The material used in this study is stored in the collection of the State Scientific Research Institute Nature Research Centre, Lithuania.

### 2.2. DNA Extraction, PCR and Sequencing

Molecular analysis was used for the identification of the parasites. DNA from bird blood samples positive for the presence of trypanosomes as detected by the buffy coat method and preserved in SET buffer was extracted using the ammonium acetate DNA precipitation protocol [39]. PCR-based screening for large-size *Trypanosoma* was carried out using outer primers, Tryp763 (5′-CATATGCTTGTTTCAAGGAC-3′) and Tryp1016 (5′-CCCCATAAT-CTCCAATGGAC-3′), and inner primers, Tryp99 (5′-TCAATCA-GACGTAATCTGCC-3′) and Tryp957 (5′-CTGCTCCTTTGT TATCCCAT-3′) [40,41,42], which amplify DNA fragment coding for the SSU 18S rRNA from *Trypanosoma* spp., as well as other trypanosomatids [27]. The PCR mix consisted of a total volume of 25 μL and contained 12.5 μL of DreamTaq PCR Master Mix (Thermo Fisher Scientific, Vilnius, Lithuania), 8.5 μL of nuclease-free water, 1 μL of each primer and 2 μL of DNA template.

Nematode DNA was extracted from blood preserved in ethanol according to Stunžėnas et al. [43], with a minor modification according to Petkevičiūtė et al. [44], or using the KAPA Express Extract Kit (KAPA Biosystems, Cape Town, South Africa) following the manufacturer’s protocols. Only samples from birds infected with a single microfilariae infection as detected using both the buffy coat method and microscopy were analysed using PCR. Partial sequences of the nuclear *28S* rDNA (*28S*) gene fragment of 765 bp were amplified using the nematode-specific primers Nematode 1 (5′-GCGGAGGAAAAGAAACTAA-3′) and Nematode 2 (5′-ATCCGTGTTTCAAGACGGG-3′) following the temperature profile as in the original protocol [45]. During amplifications, the PCR mix consisted of a total volume of 25 μL and contained 12 μL of DreamTaq PCR Master Mix (Thermo Fisher Scientific, Vilnius, Lithuania), 9 μL of nuclease-free water, 1 μL of each primer and 2 μL of DNA template.

To evaluate the amplified products for both parasites, a 1% agarose gel was used. Positive samples were precipitated using an ammonium acetate protocol [39] and sequenced using a Big Dye Terminator V3.1 Cycle Sequencing Kit and ABI PRISMTM 3100 capillary sequencing robot (applied Biosystems, Foster City, CA, USA). Geneious Prime software ver. 2023.2.1 (Biomatters, Auckland, New Zealand) was used to assemble and revise the sequences. Samples were sequenced in both directions. The obtained sequences were deposited to the GenBank.

### 2.3. Statistical Methods

Only data from bird species where at least 15 individuals were collected per single season (spring and autumn) were used for the analysis, except for the Eurasian siskin (*Spinus spinus* (L.)) of which only 9 individuals were collected in the spring (but in total, 153 Eurasian siskins collected both in spring and autumn were investigated) (Table 1). The parasitological indices (prevalence of parasites (P%)) were calculated according to Bush et al. [46]. The prevalences of parasites in different seasons; for different ecological groups of birds (long-distance and short-distance migrants as determined by Logminas [35] and Spina et al. [36]; insectivorous and omnivorous feeding behaviour and birds nesting in open nests and in nest boxes as determined by Logminas [35] and Svensson et al. [47]); and the prevalences of parasites between different years of investigation were compared using the Fisher exact test. A *p* value of less than 0.05 was considered to be significant. Juveniles of some bird species were determined [48], and the prevalences of parasites between adults and juveniles caught in autumn were compared using the same test.

## 3. Results

### 3.1. Prevalence of Parasites in Different Species of Birds

In total, 3335 birds belonging to 19 species were caught and investigated for the presence of *Trypanosoma* and Onchocercidae (Table 1). Of the tested birds, 483 (14.5%) were infected with *Trypanosoma* and 80 (2.4%) with Onchocercidae. Mixed infections of both *Trypanosoma* and microfilaria were detected in 20 birds (0.6%). All investigated bird species were infected with *Trypanosoma* parasites. The highest prevalences of *Trypanosoma* were detected in Common redstarts (*Phoenicurus phoenicurus* (L.)) (n = 61, 36.1%), dunnocks (*Prunella modularis* (L.)) (n = 80, 35.0%), Eurasian blackcaps (*Sylvia atricapilla* (L.)) (n = 133, 29.3%), goldcrests (*Regulus regulus* Sundevall, 1850) (n = 43, 25.6%) and Willow warblers (*Phylloscopus trochilus* (L.)) (n = 124, 25.0%). The lowest prevalences were characteristic of Great tits (*Parus major* L.) (n=406, 3.9%) and European starlings (*Sturnus vulgaris* L.) (n = 267, 2.2%) (Table 1). We have found bird species not infected with filaria, even though more than 100 birds belonging to the same species were investigated in many cases (Table 1). Eurasian siskins*,* Willow warblers*,* European starlings and Eurasian wrens (*Troglodytes troglodytes* (L.)) were found not to be infected with Onchocercidae. The highest prevalences of filaria were detected in Song thrushes (*T. philomelos*) (n = 98, 17.3%), blackbirds (*Turdus merula*) (n = 69, 10.1%), Eurasian blue tits (*Cyanistes caeruleus* (L.)) (n = 318, 5.0%), Common chiffchaffs (*Phylloscopus collybita* (Vieillot, 1817)) (n = 86, 4.7%) and Common chaffinches (*Fringilla coelebs* L.) (n = 180, 3.3%).

### 3.2. The Prevalence of Parasites in Spring and Autumn

Out of all the investigated birds, 1649 were caught in spring and 1686 birds were caught in autumn (Table 1). The prevalences of Onchocercidae parasites in birds were 2.2% in spring and 2.6% in autumn. No statistically significant differences between the prevalences of Onchocercidae in spring and autumn for each bird species were determined. The prevalence of Trypanosomatidae parasites in birds was 16.3% in spring and 12.7% in autumn. Statistically significant differences in prevalences of *Trypanosoma* parasites were determined for three bird species. Sedge warblers (*Acrocephalus schoenobaenus* (L.)) and barn swallows (*Hirundo rustica* L.) were found to be infected with higher prevalences in spring (13.9% and 35.2%, respectively) than in autumn (no infected birds were detected), *p* = 0.02 and *p* = 0.0001, respectively. The difference between the prevalences of *Trypanosoma* parasites in common reed warblers (*A. scirpaceus*) was close to a statistically significant value (*p* = 0.053). On the contrary, the prevalence of *Trypanosoma* parasites of *T. philomelos* was higher in autumn compared with that in spring, *p* = 0.003 (Table 1).

### 3.3. The Prevalence of Parasites in Long-Distance and Short-Distance Migrants

Based on the literature data [35,36], we divided the studied birds into long-distance (n = 1274) and short-distance (n = 2061) migrants (Supplementary Table S1) and analysed them separately. The prevalence of *Trypanosoma* parasites in long-distance migrants was 18.3% (233 infected birds), while in short-distance migrants it was 12.1% (250 infected birds), and this difference was statistically significant (*p* = 0.0001). The prevalence of Onchocercidae in long-distance migrants was 2.7% (34 infected birds) and in short-distance migrants was 2.3% (48 infected birds), and this difference was not significant (*p* = 0.57).

### 3.4. The Prevalence of Parasites in Birds with Different Diets

We analysed birds based on their diet, dividing them into insectivorous (n = 1656) and omnivorous, feeding on both insects and seeds or berries (n = 1679) (Supplementary Table S1). The prevalence of *Trypanosoma* parasites in insectivorous birds was 12.6% (208 infected birds), while in omnivorous birds it was 16.4% (275 infected birds), and this difference was statistically significant (*p* = 0.002). The prevalence of Onchocercidae in insectivorous birds was 1.1% (18 infected birds) and in omnivorous birds it was 3.8% (64 infected birds), and this difference was also statistically significant (*p* = 0.0001).

### 3.5. The Prevalence of Parasites in Open-Nesting Birds and Birds Nesting in Nest Boxes

Based on the biology of the birds, we analysed birds nesting in open nests (n = 2283) and in nest boxes (n = 1052) (Supplementary Table S1). The prevalence of *Trypanosoma* parasites in open-nesting birds was 16.9% (385 infected birds), while in birds nesting in nest boxes it was 9.3% (98 infected birds), and this difference was statistically significant (*p* = 0.0001). The prevalence of Onchocercidae in open-nesting birds was 2.5% (58 infected birds) and in birds nesting in nest boxes it was 2.3% (24 infected birds) and did not differ (*p* = 0.7).

### 3.6. Composition of Trypanosoma Parasites

**Figure 1:**
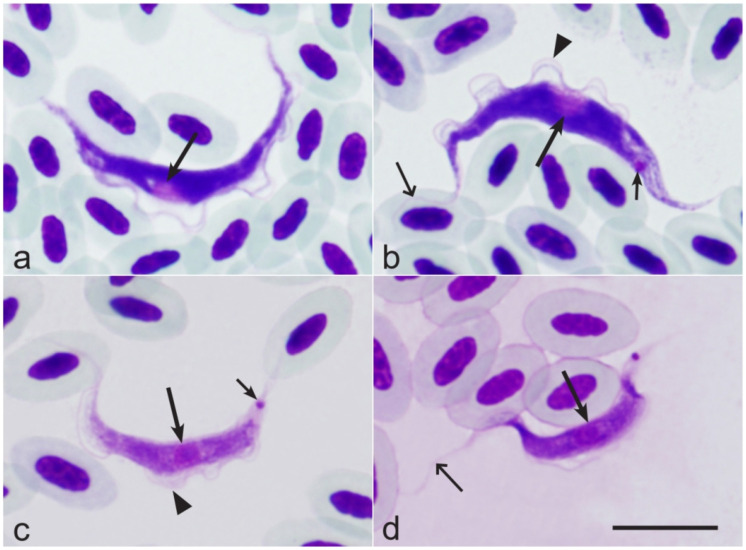
Hematozoic trypomastigotes of *Trypanosoma avium* from the Eurasian chaffinch *Fringilla coelebs* (**a**,**b**) and *Trypanosoma culicavium* from the Barn swallow *Hirundo rustica* (**c**,**d**). Long arrows—nuclei of parasites; short arrows—kinetoplast; simple wide arrows—flagellum; triangle arrowheads—undulating membrane. Giemsa-stained thin blood films. Bar = 10 µm.

We found mixed infections of both large-size and small-size *Trypanosoma* in the same bird in 9.2% of trypanosome-positive cases. We were not able to determine the mixed infection of two different groups of large trypanosomes as based on our data, as the blood stages of large-size *Trypanosoma* parasites of the *T. culicavium* and *T. avium* groups are very similar morphologically (Figure 1). *Trypanosoma culicavium* Votypka et al., 2012 was first described not from bird blood, but from mosquitoes [49]. Using PCR-based investigation, usually only one parasite can be detected from a mixed infection. The prevalences of the small-size *Trypanosoma bennetti* group and large-size *Trypanosoma* parasites in the investigated birds were similar and were 49.1% and 41.7%, respectively. PCR-based investigation of large-size *Trypanosoma* can be used for species identification. In our case, only sequences of 28.8% of investigated samples were successfully obtained using PCR and showed that birds were infected with *T. avium* Danilewsky, 188*5* as well as *T. culicavium* parasites: *T. avium* was detected in *F. coelebs, S. atricapilla,* and *Ph. Phoenicurus;* and *T. culicavium* was detected in *H. rustica, Ph. trochilus,* European robin (*Erithacus rubecula* (L.)) and *S. atricapilla* (Supplementary Table S2). We detected some statistically reliable differences in *Trypanosoma* prevalences between different years of investigation (Supplementary Table S3). During the research, we were able to identify the juveniles of some of theinvestigated bird species. Small-size trypanosomes were detected in juveniles of *C. caeruleus, S. spinus, P. major, T. merula* and *T. philomelos*. Both small and large trypanosomes were detected in *P. modularis* and *E. rubecula* juveniles. Large trypanosomes were detected in *R. regulus* juveniles. Comparing the prevalences of infection in adults and juveniles of the same species caught in autumn, the prevalence of *Cyanistes caeruleus* juveniles was found to be statistically significantly higher as compared with adults (*p* = 0.015).

### 3.7. Composition of Onchocercidae Parasites

**Figure 2:**
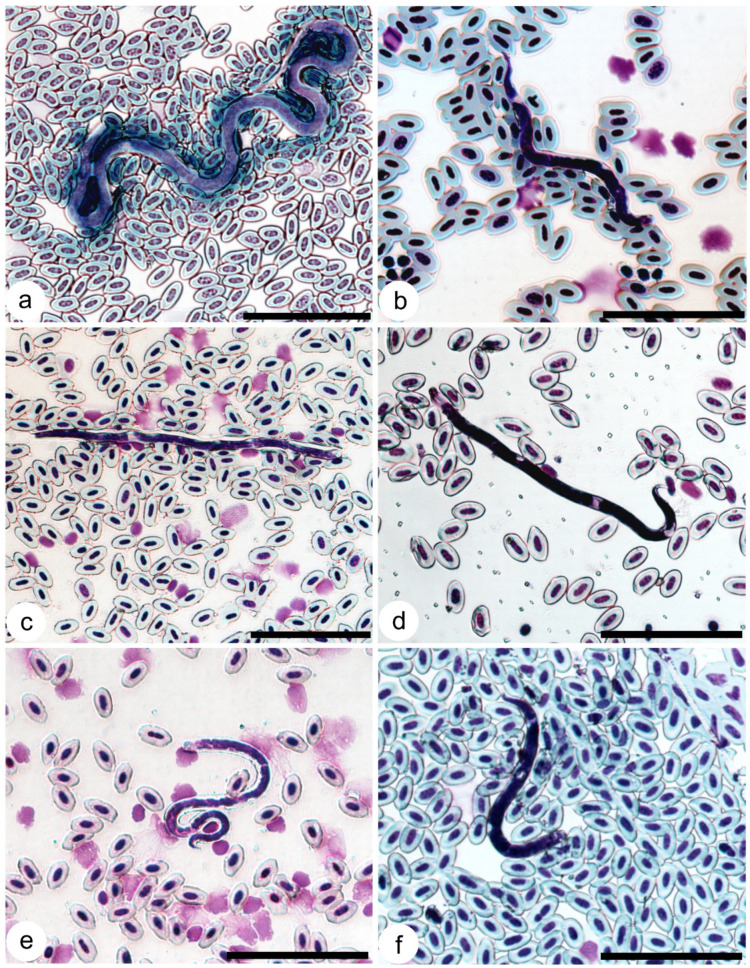
Microfilariae from bird blood: (**a**) *Cardiofilaria* sp. from the Eurasian blue tit *Cyanistes caeruleus* (HELMI1500); (**b**) *S. mavis* from the Eurasian blackbird *Turdus merula* (HELMI1413); (**c**) *Chandlerela sinensis* from the Eurasian blue tit *Cyanistes caeruleus* (HELMI1366)*;* (**d**) *Eufilaria sylviae* from the barn swallow *Hirundo rustica* (HELMI1534)*;* (**e**) *Eufilaria* sp. from the sedge warbler *Acrocephalus schoenobaenus* (HELMIS1529); (**f**) *Eufilaria* sp. 2 from the song thrush *Turdus philomelos* (HELMI1528). Giemsa-stained thin blood. Bar = 50 µm.

Three morphotypes of microfilariae were found in the investigated birds. The first morphotype was characterised by long microfilaria: length 332 µm (289–370 µm, n = 10), width 5.8 µm (5–6.4 µm, n = 10), with a long (56 µm (46–66 µm, n = 8)) sharply pointed tail (accession numbers: HELMII468-1521) (Figure 2a). These microfilariae were found in 28 birds belonging to four species (*C. caeruleus, E. rubecula, P. major* and *T. philomelos*). The sequences from these microfilariae clustered together, but did not belong to any genus of nematode for which sequences have been deposited in GenBank. The second morphotype of microfilaria can be characterised by a shorter length (up to 122 µm (79–122, n = 15)) and similar width (5.7 µm (5–6 µm, n = 15)), with a shorter (16 µm (9–19 µm, n = 15)) sharply pointed tail (accession numbers: HELMI1394-1467) (Figure 2b). This morphotype was detected in 30 birds belonging to *C. caeruleus, E. rubecula, P. major, Ph. collybita, P. modularis, T. merula, T. philomelos* species. Sequences of this type of microfilaria 100% match *Splendidofilaria mavis* sequence (GenBank: OK644715.1). The third, most common morphotype of microfilaria can be characterised by a medium length (147 µm (129–160 µm, n = 12)) and width (3.9 µm (3–5 µm, n = 12)), with a 15 µm length (12–20 µm, n = 15) broadly rounded tail and extended sheath (accession numbers: HELMI1333-1393) (Figure 2c). The third morphotype was found in 21 birds belonging to *C. caeruleus, F. coelebs, P. major, Ph. collybita, P. modularis* and *S. borin*. Sequences of this type of microfilaria match the *Chandlerella sinensis* sequence (GenBank: OR350920.1, 99.8–100% coverage). The sequences of microfilariae (Figure 2d) from *H. rustica* and *S. borin* 99.9–100% match the *Eufilaria sylviae* sequence (GenBank: MT802311.1). All sequences from the blood of *A. schoenobaenus* (Figure 2e) match 99.7–100% with *Eufilaria* sp. (GenBank: MT802310.1). We obtained sequences of microfilariae from *T. merula* and *T. philomelos* (Figure 2f) that differed from other sequences deposited in GenBank but were closest to the genus *Eufilaria*. Two specimens of *T. philomelos* were infected with microfilaria of 102 µm (91–125 µm, n = 10) in length and 5.5 µm (4–6.6 µm, n = 10) in width, with a 16 µm (11–19 µm, n = 15) broadly rounded tail, but without a sheath (accession numbers: HELMI1490-1491). We were not able to identify this parasite species using PCR as these birds had mixed infections of different microfilariae. Mixed infections of two microfilariae species were found in 17 birds (21.2% of infected birds), and one *T. philomelos* was infected with three species of microfilariae. The majority of long-distance migratory birds had a single Onchocercidae infection, except the already-mentioned *T. phillomelos* and *S. borin*. Four out of six short-distance migratory birds were infected with more than one species of microfilariae (Supplementary Table S4). Juveniles of C. *caeruleus, P. major* and *T. philomelos* were infected with long microfilariae of the first morphotype, *E. rubecula, T. merula, T. philomelos, P. phoenicurus* and *P. modularis*—*S. mavis* microfilariae, *T philomelos*—*Eufilaria* sp., and *P. modularis*—*C. sinensis* microfilariae (Supplementary Table S4). No differences in the prevalences of microfilaria have been detected between different years of investigation. Comparing the prevalences of infection in adults and juveniles of the same species caught in autumn, the prevalence of infected adults was 3.3% (11 infected birds) and in juveniles was 2.5% (20 infected birds), and this difference was not significant (*p* = 0.43).

## 4. Discussion

Our study showed that all investigated bird species were infected with trypanosomes, but the prevalences of *Trypanosoma* differed in different bird species and varied between 2.2% (*S. vulgaris*) and 36.1% (*Ph. phoenicurus*), while the prevalences of Onchocercidae parasites varied between 0 and 17.3% (*T. philomelos*). The infection rate may depend on the bird's immune system; for example, starlings seem to be extremely resistant to some parasite infections compared to other bird species [50], and this may be one of the reasons for the wide distribution of starlings. However, the possibility of infection with certain blood parasites may also depend on some ecological features of birds. For example, it was found that birds living in reedbeds have less contact with *Culicoides* Latreille, 1809 biting midges; therefore, they are less likely to be infected with *Haemoproteus* Kruse, 1890 parasites transmitted by these vectors [51]. Similarly, our studies have shown that the infection of bird species breeding in reedbeds, such as *A. schoenobaenus* and *A. scirpaceus,* is high in spring after winter migration and is very low or zero after the breeding period in autumn (Table 1). We know that parasites of the *T. bennetti* group, as well as some filariae, are transmitted by the same *Culicoides* biting midges which are known to transmit *Haemoproteus* parasites [6,27], only the mode of transmission of haemosporidian parasites and trypanosomes is different [28]. Similarly, the prevalence of *Trypanosoma* parasites in *H. rustica* was very high in spring, reaching 35.2%, and was 0% in autumn, although 133 birds were tested (Table 1). It is possible that these birds do not become infected with the parasites during breeding season, so in autumn, when many juveniles enter the trapping nets, the prevalence decreases significantly due to the dilution effect. *Trypanosoma bennetti*-group trypanosomes are transmitted experimentally by subcutaneous inoculation and may be transmitted through the direct ingestion of infected *Culicoides* biting midges in the wild [28]. *Hirundo rustica* are insectivorous birds, so it can be assumed that the birds become infected with parasites (we know that infection with the *T. bennetti* group occurs in the study area for other bird species, and it seems that these parasites are not very specific to avian hosts), but they do not have time to develop by the time the juveniles leave the breeding sites for the wintering grounds. Otherwise, more detailed studies on the diet of these birds should be conducted.

In contrast, *T. philomelos*, which were not infected in spring, were heavily infected with trypanosomes in autumn. These parasites were also found in Song thrush juveniles, indicating that birds were infected at the study site and that *T. bennetti*-group parasite transmission is occurring here. Based on our data and the fact that the detection of parasites in juveniles indicates the presence of transmission at the study site, we can state that transmission of *T. bennetti*-group parasites occur in *P. major, P. modularis, E. rubecula, C. caeruleus, S. spinus, T. merula* and *T. philomelos* in our study area. The prevalence of trypanosomes in *C. caeruleus* juveniles investigated in autumn was even higher compared with that of adults. Large-size trypanosomes, which are transmitted by black flies *(T. avium*) and *Culex* Linnaeus, 1758 mosquitoes (*T. culicavium*), were also found in the juveniles of three bird species (*R. regulus, P. modularis* and *E. rubecula*). These parasites are transmitted by vector ingestion and via conjunctiva in the case of *T. avium* [30]. The prevalences of *Trypanosoma* parasites in the majority of bird species investigated during this study was similar in both spring and autumn and did not differ statistically (Table 1).

Due to the bird’s ability to fly long distances the possibility to meet different vectors, to get parasites and to carry them to different ecozones is much higher [52]. We expected that long-distance migratory birds would be more infected compared with short-distance migrants, and this was the case for the *Trypanosoma* parasites. It is possible that long-distance migrants, travelling through different territories, face a higher risk of infection with blood parasites due to the higher diversity of parasites as well as vectors in southern regions. On the other hand, migratory birds can avoid exposure to high vector densities while migrating. However, the prevalence of infection was also high in some species of short-distance migrants, such as *S. spinus* (24.8%) or *P. modularis* (35.0%).

This study indicated that the prevalence of Onchocercidae parasites did not differ significantly between long- and short-distance migrants. However, the possibility of finding long-distance migrants infected with microfilariae is greater in spring, as seven species were found to be infected at this time, and only three species were detected to be infected in autumn. Two of these species, *Ph. collybita* and *T. philomelos*, were found to be infected with Onchocercidae parasites during both spring and autumn, but this could be because these birds may winter not only in North Africa and South Asia, but also in southern and western Europe [36] where we indicated at least four Onchocercidae species completing their life cycles. One *S. borin* bird sampled in spring was infected with *C. sinensis* microfilariae, indicating that this bird also overwintered in southern Europe. *Phoenicurus phoenicurus,* a long-distance migratory bird, was infected with onchocercidian nematodes only in autumn. During the winter, this bird migrates to tropical zone of Africa, where many bloodsucking insects are present [53], but is not infected with filarioids. We detected one juvenile Common redstart infected with *S. mavis* in autumn. This parasite can infect many short-distance migratory birds of different species. It seems that infection with this parasite occurs during breeding season, and this is one more piece of evidence that the transmission of onchocercidian parasites is taking place not only in the southern temperate zone, but in the north Eastern Baltic region as well.

The fact that the prevalence of both trypanosomes and filaria in omnivorous birds was statistically significantly higher compared with insectivorous birds was unexpected, because avian trypanosomes are transmitted to vertebrate hosts not by the bite, but by direct ingestion of an infected insect or by conjunctiva [28]. Some species of insectivorous birds were heavily infected with *Trypanosoma* (*H. rustica, Ph. phoenicurus, P. modularis, R. regulus*), but bird species whose diet characteristically includes both insects and berries or insects and seed were also heavily infected with trypanosomes. In this work, we did not include vegetarian birds, as all investigated birds more or less feed on insects. The part of infected insects in the study area seems to be important for the infection of birds as well as the diet of birds, because they can use some insects more frequently than others for food.

The prevalence of *Trypanosoma* in open-nesting birds was statistically higher compared with birds nesting in nest boxes. Some bloodsucking insects, for example black flies, avoid closed cavities and in this way were not a big threat for birds in nest boxes. Other bloodsucking insects can enter nest boxes [54], but we do not know about the difference in their activity in open space and indoors.

We did not detect differences in the prevalences of microfilaria between different years of investigation (2018-2024), but some differences in the prevalences of *Trypanosoma* parasites have been determined (Supplementary Table S3). These differences can be related to the fact that different numbers of birds of different species were caught and investigated in different years. Only in 2021-2023, birds were collected in both spring and autumn, and the differences between these three years were not significant. Our research has shown that the prevalence of certain parasites in the same host species can vary in different seasons. When studying avian blood parasites, it is necessary to pay attention not only to the host species, but also to the season.

In our study, the buffy coat method was used as the primary method to determine whether birds were infected with the parasites in this study. This is a technique developed to concentrate the parasites from investigated blood. The method is based on blood centrifugation and the resulting separation of blood cells and parasites in different layers [55]. This method is commonly used in parasitology, particularly for the detection of *Trypanosoma* and microfilariae in humans [56,57] and domestic animals [58]. This is a sensitive method that allows the detection of trypanosomes and filariae at very low parasitaemia, which is usually the case in the wild, when microscopic examination and PCR cannot be used an effective method to detect these parasites. On the other hand, this method has disadvantages for use in the field, as it needs a centrifuge, microscope and electricity, and other methods should be used in parallel in order to identify detected parasites. We used only a drop of blood (approximately 10 µl) for molecular testing, so even the PCR method was not sensitive enough and in many cases, it was not possible to obtain *Trypanosoma* sequences, although the presence of trypanosomes was confirmed by the buffy coat method.

The best way to study the onchocercidian parasites and have the smallest impact on the birds is to investigate blood stages of these parasites, as just a few drops of blood are enough to detect and identify the parasite. However, there are difficulties in working with onchocercidians due to the circadian rhythms of these parasites [23] and the morphological similarity of microfilariae of different species or even genera. Bartlett [4] tried to summarise some morphological features to distinguish at least parasite genera for the microfilaria stage. According to him, microfilariae longer than 200 µm with sharply pointed tail can be assigned to *Pelecitus*, *Struthiofilaria* Noda and Nagata, 1976 and *Cardiofilaria* genera. The genus *Struthiofilaria* has only one species which was found in an ostrich which died in Misaki Park Zoo in Japan [59]. The genus *Pelecitus* has 17 valid species, but in the European temperate zone only two species were found: *Pelecitus fulicaeatrae* (Diesing, 1861) was detected in *Fulica atra* L. (Gruiformes), and *Pelecitus chabaudi* Bartlett and Greiner, 1986 was detected in *Pernis apivorus* L. (Accipitriformes) [13,20]. The genus *Cardiofilaria* has 14 valid species and 2 of them (*Cardiofilaria pavlovskyi* Strom, 1937 and *Cardiofilaria campanae* (Chabaud and Golvan, 1956) are found in passerine birds in Europe [17,21]. Based on these facts, we can make the assumption that the long microfilaria detected during this study belong to the genus *Cardiofilaria* and most probably to the species C. *pavlovskyi,* as this species, in addition to parasitising a number of bird species, has been found in neighbouring Poland in *P. major* [60]. We found two specimens of *T. philomelos* infected with microfilaria with a broadly rounded tail, but without a sheath. We were not able to identify the species of these parasites using PCR as birds had a mixed infection of microfilariae. This type of microfilariae was indicated as *S. mavis* in previous studies of blood parasite smears [61], but phylogenetic studies revealed that *S. mavis* microfilaria have a sharply pointed tail [18], suggesting the need for studies that use both morphological and molecular tools.

As the morphological characteristics of microfilariae of different species can be similar, molecular studies would be very helpful in species description. Unfortunately, there are too few studies describing adults of avian onchocercidian parasites using both morphology and molecular markers [14,18,23,62,63]. As a result of these phylogenetic studies, *H. rustica* was found to be infected with *E. sylviae*, which had previously been described only in *S. borin* [14]. The timing of the migration of the two birds is more or less similar: both birds arrive to Lithuania at the second half of April or the beginning of May and depart in September to October, and both may fly to central Africa [36,64], where they can be infected with these parasites. Therefore, our study of avian blood parasites revealed not only the species of parasites but also the wintering sites of one *S. borin* and two *H. rustica* individuals.

## 5. Conclusions

Both *Trypanosoma* and Onchocercidae parasites are found in avian blood and can be detected using the same methods. The concentration of parasites in the blood of both groups is low enough that the buffy coat method is suitable for their detection, but identification issues pose different challenges. Both groups of parasites are transmitted by bloodsucking vectors. However, the modes of transmission of the two groups of parasites are different—*Trypanosoma* are transmitted by ingestion or by conjunctiva, and Onchocercidae by the bite of bloodsucking insect. This is the reason why the factors determining the different prevalences in birds are different. We can say that the prevalence of avian blood parasites is influenced by the diet, breeding behaviour and migration features of vertebrate hosts, the significance of which remains to be assessed. Based on infections of juveniles, we found that the transmission of trypanosomes and of some species of onchocercid occurs not only in southern Europe or Africa, but also in the northern climatic zone such as the eastern Baltic countries.

## Figures and Tables

**Table 1:** The prevalences (P%) of Onchocercidae (Mf) and *Trypanosoma* (Try) parasites during the spring and autumn of the investigated birds. N—the number of birds. Bold—differences between spring and autumn are statistically significant.

Bird Species	Spring			Autumn		
	N	Mf N (P%)	Try N (P%)	N	Mf N (P%)	Try N (P%)
*Acrocephalus schoenobaenus* (L.)	209	3 (1.4)	29 (**13.9)**	33	0	**0**
*Acrocephalus scirpaceus* (Hermann, 1804)	93	2 (2.2)	14 (15.1)	50	0	2 (4.0)
*Cyanistes caeruleus* (L.)	90	4 (4.4)	15 (16.7)	228	12 (5.3)	39 (17.1)
*Erithacus rubecula*	174	4 (2.3)	12 6.9)	261	4 (1.5)	20 (7.7)
*Fringilla coelebs* L.	138	6 (4.3)	31 (22.5)	42	0	8 (19.0)
*Hirundo rustica* (L.)	142	2 (1.4)	50 (**35.2)**	133	0	**0**
*Parus major* L.	118	1 (0.8)	4 (3.4)	288	6 (2.1)	12 (4.2)
*Phoenicurus phoenicurus* (L.)	24	0	10 (41.7)	37	1 (2.7)	12 (32.4)
*Phylloscopus collybita* (Vieillot, 1817)	32	1 (3.1)	5 (15.6)	54	3 (5.6)	11 (20.4)
*Phylloscopus trochilus* (L.)	107	0	28 (26.2)	17	0	3 (17.6)
*Prunella modularis* (L.)	38	0	10 (26.3)	42	2 (4.8)	18 (42.9)
*Regulus regulus* Sundevall, 1850	19	0	5 (26.3)	24	0	6 (25.0)
*Spinus spinus* (L.)	9	0	1 (11.1)	144	0	37 (25.7)
*Sturnus vulgaris* L.	218	0	5 (2.3)	49	0	1 (2.0)
*Sylvia atricapilla* (L.)	101	2 (2.0)	34 (33.7)	32	0	5 (15.6)
*Sylvia borin* (Boddaert, 1783)	60	3 (5.0)	5 (8.3)	52	0	2 (3.8)
*Troglodytes troglodytes* (L.)	24	0	3 (12.5)	86	0	11 (12.8)
*Turdus merula* L.	33	4 (12.1)	8 (24.2)	36	3 (8.3)	4 (11.1)
*Turdus philomelos* Brehm, 1831	20	5 (25.0)	**0**	78	12 (15.4)	23 (**29.5)**
Total	1649	37 (2.2)	269 (16.3)	1686	43 (2.6)	214 (12.7)

## Data Availability

All data generated or analysed during this study are included in this published article. All newly generated sequences were submitted to the GenBank database (accession numbers: PV454181-PV454205). The type and voucher material (see parasite descriptions) were deposited in the State Research Institute Nature Research Centre, Lithuania (accession numbers: HELMI1333-1545).

## Supplementary Materials

The following supporting information can be downloaded at: https://www.mdpi.com/article/10.3390/pathogens14050452/s1. The online version contains four supplementary tables. Additional File S1: Table S1. Investigated bird species, numbers of birds, migration patterns, diet, nesting behavior. Additional File S2: Table S2. *Trypanosoma* parasites detected in long-distance and short-distance passerine birds. Additional File S3: Table S3. Differences (*p* values) in prevalences of *Trypanosoma* parasites in birds in different years of investigation. Additional File S4: Table S4. Onchocercidian nematodes species found in long-distance and short-distance passerine birds.

## Abbreviations

The following abbreviations are used in this manuscript:

cox1	Mitochondrial cytochrome c oxidase I
µm	Micrometres
µL	Microlitres
bp	Base pair
DNA	Deoxyribonucleic acid
