# Supplementary material for: Neglected Avian Blood Parasites (Onchocercidae and Trypanosomatidae) in Migratory Passerines of the Temperate Zone, Eastern Baltic Region
Source: Pathogens. 2025 May 5;14(5):452. doi: 10.3390/pathogens14050452 (PMC12114413; doi:10.3390/pathogens14050452)
Supplement: Supplementary file 1 [file pathogens-14-00452-s001.zip › Supplementary Table S1.pdf]

Table S1. Investigated birds, their numbers, migration patterns, diet, nesting behavior.

Bird species	Bird common name	N	Migration	Diet	Nest
**Acrocephalidae**					
Acrocephalus schoenobaenus	Sedge warbler	242	LD	I	Op
Acrocephalus scirpaceus	Common reed warbler	143	LD	I	Nb
**Fringillidae**					
Fringilla coelebs	Common chaffinch	180	SD	IS	Op
Spinus spinus	Eurasian siskin	153	SD	IS	Op
**Hirundinidae**					
Hirundo rustica	Barn swallow	275	LD	I	Op
**Muscicapidae**					
Erithacus rubecula	European robin	435	SD	I	Op
Phoenicurus phoenicurus	Common redstart	61	LD	I	Nb
**Paridae**					
Cyanistes caeruleus	Eurasian blue tit	318	SD	IS	Nb
Parus major	Great tit	406	SD	IS	Nb
**Phylloscopidae**					
Phylloscopus collybita	Common chiffchaff	86	LD	I	Op
Phylloscopus trochilus	Willow warbler	124	LD	I	Op
**Prunelidae**					
Prunella modularis	Dunnock	80	SD	I	Op
**Regulidae**					
Regulus regulus	Goldcrest	43	SD	I	Op
**Sturnidae**					
Sturnus vulgaris	European starling	267	SD	I	Nb
**Sylviidae**					
Sylvia atricapilla	Eurasian blackcap	133	LD	IB	Op
Sylvia borin	Garden warbler	112	LD	IB	Op
**Troglodytidae**					
Troglodytes troglodytes	Eurasian wren	110	SD	I	Op
**Turdidae**					
Turdus merula	Eurasian blackbird	69	SD	IB	Op
Turdus philomelos	Song thrush	98	LD	IB	Op

N – numbers of infected birds, LD – long-distance migratory bird, SD – short-distance migratory bird, I – insectivorous birds, IS – insects and seeds eating birds, IB – insects and berries eating birds, Nb – nest in nest box or other enclosed spaces such as tree hollows, Op – nest in open area, Ma – March, Ap – April, My – May, Jn – June, Jl – July, Au – August.
