# Supplementary material for: Neglected Avian Blood Parasites (Onchocercidae and Trypanosomatidae) in Migratory Passerines of the Temperate Zone, Eastern Baltic Region
Source: Pathogens. 2025 May 5;14(5):452. doi: 10.3390/pathogens14050452 (PMC12114413; doi:10.3390/pathogens14050452)
Supplement: Supplementary file 1 [file pathogens-14-00452-s001.zip › Supplementary Table S2.pdf]

Table S2. *Trypanosoma* parasites detected in long-distance and short-distance passerine birds.

Bird species	Spring		Autumn	
Trypanosoma group	*T. everetti* gr.	*T. avium /culicavium* gr.	*T. everetti* gr.	*T. avium /culicavium* gr.
**Long-distance**				
Acrocephalus schoenobaenus	+	+ (*T. culicavium*)	NI	NI
Acrocephalus scirpaceus	+	+	+	+
Hirundo rustica	+	+ (*T. culicavium*)	NI	NI
Phoenicurus phoenicurus	+	+ (*T. avium*)	+	+
Phylloscopus collybita	+	+	+	+
Phylloscopus trochilus	+	+ (*T. culicavium*)	+	+
Sylvia atricapilla	+	+ (*T. culicavium, T. avium*)	+	+ (*T. culicavium)*
Sylvia borin	NI	+	+	NI
Turdus philomelos	NI	NI	+	NI
**Short-distance**				
Cyanistes caeruleus	+	+	+	+
Erithacus rubecula	+	+ (*T. culicavium*)	+	+ (*T. culicavium*)
Fringila coelebs	+	+ (*T. avium*)	+	+ (*T. avium*)
Parus major	NI	+	+	+
Prunella modularis	+	+	+	+
Regulus regulus	+	+ (*T. culicavium*)	NI	+
Spinus spinus	NI	+ (*T. avium*)	+	+
Sturnus vulgaris	+	+	+	NI
Turdus merula	+	NI	+	+
Troglodytes troglodytes	+	+	+	+

NI – not infected.
