# Supplementary material for: Neglected Avian Blood Parasites (Onchocercidae and Trypanosomatidae) in Migratory Passerines of the Temperate Zone, Eastern Baltic Region
Source: Pathogens. 2025 May 5;14(5):452. doi: 10.3390/pathogens14050452 (PMC12114413; doi:10.3390/pathogens14050452)
Supplement: Supplementary file 1 [file pathogens-14-00452-s001.zip › Supplementary Table S3.pdf]

Table S3. Differences (P values) in prevalences of *Trypanosoma* parasites in investigated birds in different years of investigation. X – difference was not significant, **bold** – birds were investigated only in spring, underlined - birds were investigated only in autumn, **bold and underlined** - birds were investigated both in spring and autumn.

	**2019**	2020	**2021**	**2022**	**2023**	**2024**
**2018**	0.013	x	0.001	0.003	0.003	
**2019**		0.001	0.001	0.001	0.001	0.001
2020			0.001	0.011	0.014	
**2021**				x	x	0.010
**2022**					x	x
**2023**						x
