# Supplementary material for: Neglected Avian Blood Parasites (Onchocercidae and Trypanosomatidae) in Migratory Passerines of the Temperate Zone, Eastern Baltic Region
Source: Pathogens. 2025 May 5;14(5):452. doi: 10.3390/pathogens14050452 (PMC12114413; doi:10.3390/pathogens14050452)
Supplement: Supplementary file 1 [file pathogens-14-00452-s001.zip › Supplementary Table S4.pdf]

Table S4. Onchocercidian nematodes species found in long-distance and short-distance passerine birds and Gen Bank numbers.

Bird species	Spring		Autumn		GenBank
	Parasite species	%	Parasite species	%	
**Long-distance**					
Acrocephalus schoenobaenus	*Eufilaria* sp.	1.4	NI		**PV454199- PV454201**
Acrocephalus scirpaceus	*Eufilaria acrocephalusi*	2.2	NI		MT802309.1 (Binkienė et al. 2021)
Hirundo rustica	*Eufilaria sylvae*	1.4	NI		**PV454181, PV454182**
Phoenicurus phoenicurus	NI		*Splendidofilaria mavis*	2.7	**PV454186**
Phylloscopus collybita	*Chandlerela sinensis*	3.1	*C. sinensis*	3.0	**PV454204**
Phylloscopus trochilus	NI		NI		
Sylvia atricapilla	*Splendidofilaria bartletti*	2.0	NI		
Sylvia borin	*Eufilaria sylvae*	2.0	NI		
	C. sinensis	1.0			
Turdus philomelos	*S. mavis*	25.0	*S. mavis*	35.0	**PV454188- PV454192, PV454197,**
	Cardiofilaria sp.	1.3	*Cardiofilaria* sp.	6.4	**PV454198**
			Eufilaria sp. 2	3.8	
**Short-distance**					
Cyanistes caeruleus	*C. sinensis*	1.1	*C. sinensis*	2.6	**PV454187, PV454194, PV454195,**
	Cardiofilaria sp.	4.4	*S. mavis*	0.9	**PV454203**
			Cardiofilaria sp.	4.4	
Erithacus rubecula	*S. mavis*	1.2	*S. mavis*	0.8	**PV454183, PV454184**
	Cardiofilaria sp.	0.6	*Cardiofilaria* sp.	0.4	
Fringila coelebs	*C. sinensis*	4.3	NI		**PV454202**
Parus major	*Cardiofilaria* sp.	0.8	*S. mavis*	1.4	**PV454185, PV454193**
			Cardiofilaria sp.	2.1	
Prunella modularis	NI		*C. sinensis*	2.4	**PV454205**
			S. mavis	2.4	
			Cardiofilaria sp.	2.4	
Regulus regulus	NI		NI		
Spinus spinus	NI		NI		
Sturnus vulgaris	NI		NI		
Turdus merula	*S. mavis*	12.1	*S. mavis*	2.7	OK644716 (Chagas et al. 2021)
			Eufilaria sp. 2	2.7	
Troglodytes troglodytes	NA		NA		

NI – not infected; % – prevalence of parasites infection; in bald – newly deposited sequences; AN – accession numbers of bird blood slides deposited in the collection of the State Research Institute Nature Research Centre from which sequences were obtained; Binkienė et al. 2021 – Binkienė, R.; Chagas, C. R. F.; Bernotienė, R.; Valkiūnas, G. Molecular and morphological characterization of three new species of avian Onchocercidae (Nematoda) with emphasis on circulating microfilariae. 2021. *Parasites & Vectors*, 14, 137. https://doi.org/10.1186/s13071-021-04614-8; ; Chagas et al. 2021 – Chagas, C.R.F.; Binkienė, R.; Valkiūnas, G. Description and molecular characterization of two species of avian blood parasites, with remarks on circadian rhythms of avian haematozoa infections. *Animals*, 2021, 11, 3490. https://doi.org/10.3390/ani11123490.
